# Neuroendocrine response to diclofenac in healthy subjects: a pilot study

**DOI:** 10.1007/s40618-023-02118-z

**Published:** 2023-05-27

**Authors:** E. Varaldo, M. Sibilla, F. Bioletto, D. Cuboni, N. Prencipe, C. Bona, M. Ferrari, F. Viglino, L. S. Aversa, S. Grottoli, E. Ghigo, V. Gasco, A. M. Berton

**Affiliations:** Division of Endocrinology, Diabetology and Metabolism, Department of Medical Sciences, University of Turin, C.So Dogliotti 14, 10126 Turin, Italy

**Keywords:** Arginine-vasopressin, Copeptin, MR-proADM, MR-proANP, NSAIDs, Prostaglandins

## Abstract

**Purpose:**

The precise effects of non-steroidal anti-inflammatory drugs on the neuroendocrine hydro-electrolytic regulation are not precisely understood. The aim of this pilot study was to evaluate, in healthy subjects, the neuroendocrine response of the antidiuretic system to intravenous diclofenac infusion.

**Methods:**

For this single-blinded, cross-over study, we recruited 12 healthy subjects (50% women). Test sessions were divided into three observation times (pre-test; test; 48 h post-test), which were repeated equally on two different occasions, with the administration of diclofenac (75 mg in saline solution 0.9% 100 cc) on 1 day, or placebo (saline solution 0.9% 100 cc) on another day. The night before the test the subjects were asked to collect a salivary cortisol and cortisone sample, which was repeated on the night of the procedure session. Serial urine and blood samples were collected on the test day (for osmolality, electrolytes, ACTH, cortisol, copeptin, MR-proADM, MR-proANP; the last three represent more stable and analytically reliable molecules than their respective active peptides). Moreover, the subjects were evaluated with the bioimpedance vector analysis (BIVA) before and after the test. Forty-eight hours after the end of the procedure urine sodium, urine potassium, urine osmolality, serum sodium and copeptin were revaluated together with BIVA.

**Results:**

No significant changes in circulating hormone levels were observed; anyway, 48 h after diclofenac, BIVA showed a significant water retention (*p* < 0.00001), especially in extracellular fluid (ECF) (16.47 ± 1.65 vs 15.67 ± 1.84, *p* < 0.001). Salivary cortisol and cortisone tended to increase only the night after placebo administration (*p* = 0.054 cortisol; *p* = 0.021 cortisone).

**Conclusion:**

Diclofenac resulted in an increased ECF at 48 h, but this phenomenon seems to be associated with a greater renal sensibility to the action of vasopressin rather than with an increase in its secretion. Moreover, a partial inhibitory effect on cortisol secretion can be hypothesized.

**Supplementary Information:**

The online version contains supplementary material available at 10.1007/s40618-023-02118-z.

## Introduction

Non-steroidal anti-inflammatory drugs (NSAIDs) are amongst the most widely prescribed drugs for the treatment of acute pain, rheumatic diseases and inflammatory states of various origin. NSAIDs inhibit cyclooxygenases (COX), leading to a decrease in the production of prostaglandins (PGs), which in turn play a major role in inflammatory pathways [1].

Their use is not free of adverse effects; alongside the well-known and characterized renal and gastric effects, they have also been associated with the onset of the syndrome of inappropriate antidiuresis (SIAD). The risk of SIAD is higher in frail patients, with many comorbidities and impaired renal function, but it can also present in healthy subjects [2].

The effects of acute administration of NSAIDs on neuroendocrine regulation of the hydro-electrolytic balance are not precisely understood.

The role of PGs in the renal principal cells has been investigated, leading to the hypothesis that PGE2 antagonizes the antidiuretic effect of arginine-vasopressin (AVP) by binding to the basolateral E-prostanoid (EP) 1 and 2 receptors and causing a retrieval of aquaporin 2 (AQP2) from the apical side, thus favoring electrolyte-free water clearance [3]. Moreover, certain NSAIDs have been demonstrated to induce a great shift of AQP2 from intracellular vesicles to the plasma membrane, enhancing the antidiuretic effect [4].

As far as the hypothalamic pituitary adrenal (HPA) axis is concerned, an in vitro study suggested that AVP and corticotropin releasing hormone (CRH) increase the synthesis of PGE2, which in turn decreases their ability to stimulate the production of adrenocorticotropic hormone (ACTH) [5].

Evidence about the interaction between NSAIDs and atrial natriuretic peptide (ANP) is conflicting. In healthy women diclofenac reduced the amount of PGs excreted in the urine after an acute volume expansion, but it did not alter ANP levels nor natriuresis if compared to placebo [6]. On the other hand, in a model of dogs affected by heart failure, indomethacin reduced renal natriuretic response to ANP [7].

Finally, in recent years, attention has increased on the role of adrenomedullin (ADM) in cardiovascular endocrine regulation [8]. ADM is a 52-amino acid peptide mainly synthesized and secreted by endothelial and smooth muscle cells with a predominant autocrine and paracrine role at the vascular level. In the kidney, it promotes natriuresis (mostly by interfering with sodium resorption) while its effect on the urine output has shown conflicting data [8]. However, the possible interaction between NSAIDs and plasma levels of ADM is not known to date.

As the evidence is inconclusive and not rarely derived from animal or in vitro studies, new in vivo observations are needed to better understand the effects of COX inhibition on hydro-electrolytic regulating systems. All this considered, the primary outcome of this pilot study was to evaluate, in healthy subjects, the neuroendocrine response of the antidiuretic system together with possible modifications in the ECF analyzed with bioimpedance vector analysis (BIVA). In the same way, secondary outcomes were to evaluate possible independent fluctuations in mid-regional pro-atrial natriuretic peptide (MR-proANP), mid-regional pro-adrenomedullin (MR-proADM) and the HPA axis to acute intravenous diclofenac infusion.

## Subjects and methods

### Study design and procedure

This prospective single-blinded, cross-over study (ClinicalTrials.gov ID: NCT05188131) was conducted at the Division of Endocrinology, Diabetology and Metabolism of the University Hospital “City of Health and Science of Turin” (Turin, Italy) between October and December 2021. The study was approved by the Local Ethics Committee (cod. 0096442, September 21st, 2021) and was in accordance with the principles of the Declaration of Helsinki. Written informed consent was obtained from all study participants.

We recruited 12 healthy subjects, 6 men and 6 women, eligible if they were aged 20–50 years old with a BMI between 18.5 and 25 kg/m^2^.

Exclusion criteria were as follows: history of gastric disease or peptic ulcer, previous gastro-intestinal bleeding or hemorrhagic diathesis, any ongoing acute pathologic processes or current pharmacologic treatment (including oral contraceptives), NSAIDs or acetyl-salicylic documented allergy, pregnancy or breastfeeding.

All participants underwent two test sessions, with an interval of at least 3 weeks: the drug (diclofenac 75 mg, diluted in normal saline solution 0.9% 100 cc) was administered intravenously (iv) in 15’ on 1 day and placebo (normal saline 100 cc) was infused iv in 15’ on another day. In female subjects, all evaluations were carried out in the follicular phase (within 10 days from the start of menstrual flow). In the week preceding the day of the procedure, subjects were asked to drink 1.5–2 L of water per day, with a controlled diet with 3–5 g of salt per day. On the morning of the test, all participants were fasting and not drinking for at least 8 and 2 h, respectively. Moreover, for the entire duration of the testing session all participants were not allowed to drink or eat.

The test session was divided into three observation times and, as a cross-over design, the same procedure and measurements were repeated identically in all the subjects on both occasions.

Day 0: subjects had to collect a 24 h urine sample (for fluid balance) and a salivary sample for the determination of cortisol and cortisone between 11 pm and 12am.

Day 1: on procedure day (between 7.30 and 8 am), subjects were evaluated at baseline with BIVA: height and weight were measured, and resistance (Rz), reactance (Xc) and phase angle (PhA) were registered; a fasting blood sample for MR-proADM, sodium (s-Na), copeptin, MR-proANP, ACTH and cortisol was drawn. A urine sample for osmolality (u-Osm), sodium (u-Na) and potassium (u-K) was collected as well. The drug or placebo was then administered and serial blood samples for s-Na and copeptin were drawn at times + 15’, + 30’, + 45’, + 60’, + 90’, + 120’ and + 240’. At each time point a second blood sample was taken and immediately centrifuged in order to extract serum and plasma. Thus, an aliquot of such materials was stored at − 80 °C for the subsequent determination of a simplified biochemical profile, including ACTH, cortisol, MR-proANP and MR-proADM. At the end of the test, subjects were re-evaluated with BIVA and another urine sample for u-Na, u-K and u-Osm was collected. Thereafter a new 24 h urine collection was started and at night subjects were asked to take a second salivary sample for the determination of cortisol and cortisone.

Day 3: 48 h after the test, all subjects were revaluated with BIVA and another urine sample for u-Na, u-K and u-Osm and a new blood sample for s-Na and copeptin were collected.

### Laboratory measurements

*Copeptin, MR-proADM and MR-proANP*: blood from an EDTA-containing tube was centrifuged at 4000 rpm for 5’ and a plasma aliquot was immediately frozen and stored at − 80 °C until analysis. Copeptin (pmol/L), MR-proADM (nmol/L) and MR-proANP (pmol/L) concentrations were then determined with the B.R.A.H.A.M.S. KRYPTOR compact PLUS (ThermoFisher Scientific, Hennigsdorf, Germany) automated method using the TRACE (Time-Resolved Amplifed Cryptate Emission) technique. The limit of detection (LOD) of the assay is 0.9 pmol/L for copeptin, 0.05 pmol/L for MR-proANP and 0.05 nmol/L for MR-proADM; intra- and inter-assay coefficients of variation were, respectively, < 7% and < 12% for copeptin and < 4% and < 11% for both MR-proANP and MR-proADM.*p-Osm and u-Osm*: the measurements of osmolality were performed by an automatic osmometer (Osmo Station OM-6050, ARKRAY Global, Kyoto, Japan) adopting the freezing point depression method as the measurement principle. LOD is 0 mOsm/kg with an intra-assay coefficient of variation < 1%.

*ACTH and cortisol*: ACTH (ng/L) levels were determined on plasma from EDTA test tubes, using chemiluminescent immunological methods (CLIA) on the LIAISON Analyzer platform (DiaSorin, Saluggia TO, Italy), whose sensibility was 1.6 ng/L; the intra-assay and inter-assay coefficient of variation ranged up to 4.9 and 8.8%, respectively. Serum cortisol levels (μg/L) were determined by a competitive electro-chemiluminescence immunoassay automated on Cobas e601 instrument (Roche Diagnostics GmbH, Germany). Analytical sensibility was 0.18 μg/L. Intra- and inter-assay precision ranged from 3.0 to 5.7% and from 2.4 to 6.2%, respectively.

*Salivary cortisol and cortisone*: salivary samples were collected using Salivette^®^ (SARSTEDT, Nümbrecht, Germany). Cortisol and cortisone were determined using a LC–MS/MS analysis with the MassChrom^®^ kit (Chromsystems Instruments & Chemicals GmbH, Gräfelfng, Germany). Nexera X2 UHPLC system (Shimadzu, Kyoto, Japan) was used for quantification, coupled with a triple-quadrupole mass spectrometer 4500MD (AB Sciex, Framingham, MA, USA). The sensibility for salivary cortisol is 0.28 µg/L, the intra- and inter-assay variation coefficient is 5.5 and 8.8%, respectively; cortisone sensibility is 0.55 µg/L, with intra- and inter-assay variation coefficients of 4.9 and 8.8%, respectively. Patients were instructed to soak the sample for 2–3’ and then put it in the plastic container at + 4 °C. Samples were collected at least 30’ before eating or drinking to avoid any contamination and patients were asked to have their teeth brushed at least 30’ before sampling. Smoking or eating licorice was prohibited and to ensure a valid sample collection, written instructions were given to patients.

*Other biochemical analysis*: every other routine biochemical determination was carried out through validated and automatized procedures in central Baldi&Riberi laboratory at the University Hospital "City of Health and Science of Turin".

#### BIVA

BIVA was evaluated by an impedance vector analyzer with measurement frequency of 50 kHz ± 1% (BIA101BIVA^®^, Akern, Loc. Montacchiello, Pisa, Italy). Both bioimpedance parameters (Rz and Xc) were normalized according to the patients’ height (H) and plotted on a Rz/H and Xc/H graph (Biavector, Bodygram Plus^®^ version 1.31). BIVA is a non-invasive technique that allows a reliable and reproducible assessment of the distribution of body fluids in several clinical settings [9], Rz reflecting conductivity through ionic solutions, Xc the impedance due to the membrane capacitance of metabolically active cells; finally, PhA represents a derived parameter, which expresses the ratio between intra- and extracellular fluid volumes. Furthermore, Biavector allows to compare the variations between repeated measurements on the same subject with the normal sex-specific ellipses of the general healthy population [10, 11]. Reliable thresholds for both overhydration and dehydration conditions have been previously identified at the lower and upper poles of the 75th sex-specific tolerance ellipse, respectively [11].

### Statistical analysis

Shapiro–Wilk test was used to assess for normality. Normal variables were expressed as mean ± standard deviation. Non-normal variables that could be normalized after logarithmic transformation were then expressed as geometric mean and interquartile range. Unnormalisable variables were expressed as median and interquartile range.

Student’s t-test for paired samples or ANOVA test for repeated measurements were used to identify longitudinal differences in variables with a normal distribution. Wilcoxon and Friedman tests were used to identify differences in median values for variables that had a non-normal distribution.

To compare the profile of the analytes during infusion of placebo or diclofenac, another method was used, with the aim of comparing the trend during the whole test, rather than each single time of observation. A curve was plotted for the parameters of each subject in the two different experimental conditions and then the area under the curve (AUC) was calculated. A t-test for paired samples was then conducted between the AUC during placebo and the AUC during diclofenac.

With respect to BIVA, mean Biavectors’ displacement between consecutive observations was evaluated with the paired one-sample Hotelling's T2 test [10].

As this was a proof-of-concept study, no preliminary analysis for sample size and power were conducted. A cut-off *p* value < 0.05 was considered as statistically significant. Statistical analysis was performed using MedCalcTM^®^ (Statistical Software version 18.11.3, MedCalc Software Ltd, Ostend, Belgium). Graphs and figures were realized with GraphPad PrismTM^®^ (version 8.01; GraphPad Software Inc., La Jolla, California).

## Results

### Patient characteristics

Between October 1^st^ and December 1^st^ 2021, 12 subjects were recruited (M/F 1/1). Median age at recruiting time was 25.5 [24–32.5] years old and the weight was normal (BMI 21 ± 1.4 kg/m^2^).

### Biochemical analytes

**Fig. 1:**
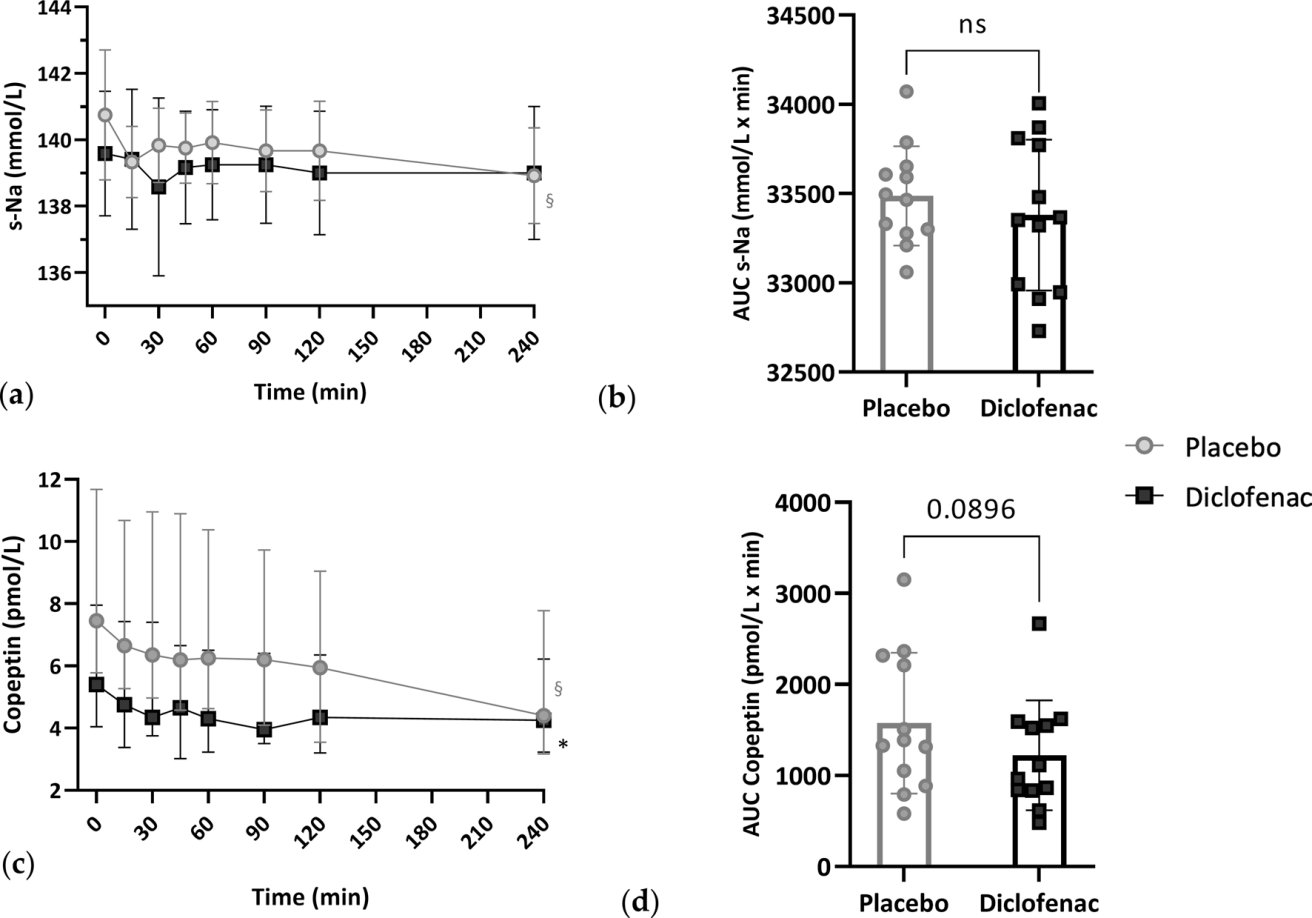
Serum sodium **a** and copeptin **c** trend during placebo and diclofenac infusion. The area under the curve (AUC) **b** and **d** was calculated for each subject and then compared with Student’s t-test. *s-Na* serum sodium, *ns* non-significant. §*p* = 0.011 for placebo profile (image **a**); §*p* = 0.002 for placebo profile; *p < 0.001 for diclofenac profile (image **c**)

*s-Na:* during placebo infusion, s-Na levels decreased significantly (*p* = 0.011), reaching the lowest level at 240’ (T0 vs T240: 140.75 ± 1.96 vs 138.92 ± 1.44 mmol/L, p = 0.034). During diclofenac, s-Na levels remained stable. When compared directly, no difference was detected in s-Na trend under the two different experimental conditions (Table 1 and Fig. 1 a-b). At 48 h, s-Na was lower than T0 after placebo (139.00 ± 1.60 mmol/L, *p* = 0.001) but not after diclofenac.

**Table 1:** Blood parameters at different observation times in the two experimental conditions

Variable	T0	T15	T30	T45	T60	T90	T120	T240	*p* value	T48h
Placebo profile										
s-Na (mmol/L)	140.75 ± 1.96	139.33 ± 1.07	139.83 ± 1.11	139.75 ± 1.06	139.92 ± 1.24	139.67 ± 1.23	139.67 ± 1.50	138.92 ± 1.44	0.011	139.00 ± 1.60
Copeptin (pmol/L)	7.45 [5.85–11.50]	6.65 [5.45–10.65]	6.35 [5.15–10.80]	6.20 [4.85–10.60]	6.25 [4.85–10.35]	6.20 [4.20–9.55]	5.95 [3.70–8.90]	4.40 [3.25–7.65]	< 0.001	5.55 [4.15−6.90]
MR-proADM (nmol/L)	0.33 ± 0.07	–	0.34 ± 0.07	–	0.35 ± 0.07	0.34 ± 0.08	–	–	NS	–
MR-proANP (pmol/L)	47.71 [36.01–49.83]	–	38.17 [31.45–45.06]	–	40.39 [33.23–47.08]	38.78 [28.89–42.89]	–	–	0.003	–
ACTH (ng/L)	22.96 [15.39–36.12]	–	14.03 [11.20–17.47]	–	12.30 [10.05–15.40]	12.51 [11.05–14.22]	–	–	0.001	–
Cortisol (μg/L)	125.75 ± 35.08	–	88.30 ± 30.23	–	73.50 ± 24.36	66.17 ± 27.24	–	–	< 0.001	–
Diclofenac profile										
s-Na (mmol/L)	139.58 ± 1.88	139.42 ± 2.11	138.58 ± 2.68	139.17 ± 1.70	139.25 ± 1.66	139.25 ± 1.76	139.00 ± 1.86	139.00 ± 2.00	NS	140.17 ± 1.64
Copeptin (pmol/L)	5.40 [4.10–7.90]	4.75 [3.55–7.35]	4.35 [3.80–7.30]	4.65 [3.15–6.60]	4.30 [3.25–6.40]	3.90 [3.60–6.30]	4.35 [3.20–6.30]	4.25 [3.35–6.15]	< 0.001	4.65 [3.45–6.60]
MR-proADM (nmol/L)	0.35 ± 0.08	–	0.33 ± 0.07	–	0.29 ± 0.07	0.31 ± 0.10	–	–	NS	–
MR-proANP (pmol/L)	37.51 [28.10–57.94]	–	35.41 [29.00–45.41]	–	36.77 [30.24–42.99]	36.77 [30.24–42.99]	–	–	NS	–
ACTH (ng/L)	21.47 ± 9.91	–	13.95 ± 6.48	–	16.74 ± 7.60	16.74 ± 7.60	–	–	0.012	–
Cortisol (μg/L)	153.75 ± 23.97	–	110.00 ± 28.25	–	93.92 ± 33.10	93.92 ± 33.10	–	–	< 0.001	–

Normal variables are expressed as mean ± standard deviation; non-normal variables that can be normalized after logarithmic transformation, are expressed as geometric mean and interquartile range; non-normal and unnormalisable variables are expressed as median and interquartile range. *p* values refer to ANOVA or Friedman test between T0 and T240 or between T0 and T90. *s-Na* serum sodium, *MR-proADM*, mid-regional pro-adrenomedullin, *MR-proANP* mid-regional pro-atrial natriuretic peptide, *ACTH* adrenocorticotropic hormone; *NS* non-significant

*Copeptin*: during placebo infusion, copeptin decreased (*p* < 0.001) progressively until 240’, when it reached its lowest level. During diclofenac, copeptin exhibited a diminishing trend (*p* < 0.001) too, with the lowest value reached at 90’ (3.90 [3.60–6.30] pmol/L). No differences emerged when the trends were compared directly (Table 1 and Fig. 1 c-d). At 48 h, copeptin was lower than T0 after placebo (T0 vs T48h: 7.45 [5.85–11.50] vs 5.55 [4.15–9.90] pmol/L, *p* = 0.042) but not after diclofenac.

In consideration of the results obtained regarding copeptin and s-Na a simplified analysis profile was performed with regard to MR-proADM, MR-proANP, ACTH and cortisol; such analytes were then evaluated just at + 30’, + 60’ and + 90’ (four timepoints).

*MR-proADM*: in both experimental conditions, it remained stable. There was no difference when the two conditions were compared directly (Table 1 and Supplementary Fig. 1 a-b).

*MR-proANP*: during saline infusion, it decreased significantly (*p* = 0.003); during diclofenac its levels did not change. However, when compared directly, the trends were comparable and no significant difference could be appreciated (Table 1 and Supplementary Fig. 1 c-d).

*ACTH*: it showed a decreasing trend during the profile in both conditions (*p* = 0.001 placebo, *p* = 0.012 diclofenac) but at a direct comparison, the two trends did not show any significant difference (Table 1 and Supplementary Fig. 2 a-b).

*Cortisol*: it decreased in both experimental conditions (*p* < 0.0001) during the test. The AUC method could not be used for cortisol, as baseline values in the two conditions were significantly different (*p* = 0.013) (Table 1 and Supplementary Fig. 2 c).

**Fig. 2:**
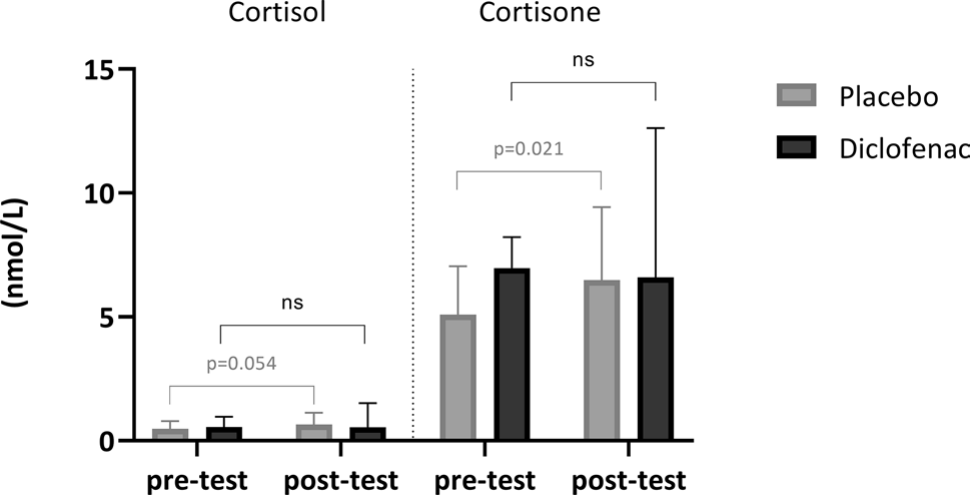
Salivary cortisol and cortisone before and after placebo and diclofenac infusion. ns: non-significant

*Salivary cortisol*: even though just above the significance level, it tended to increase after placebo administration (0.48 [0.32–0.78] vs 0.66 [0.47–1.12] µg/L, *p* = 0.054) while its levels were not altered by diclofenac (Table 2 and Fig. 2).

**Table 2:** Salivary parameters before and after the test in the two experimental conditions

Variable	Pre-test	Post-test	*p* value	Pre-test	Post-test	*p* value
	Placebo profile			Diclofenac profile		
Cortisol (nmol/L)	0.48 [0.32–0.78]	0.66 [0.47–1.12]	0.054	0.55 [0.48–0.92]	0.54 [0.41–1.36]	NS
Cortisone (nmol/L)	5.09 [4.23–6.79]	6.48 [5.29–9.00]	0.021	6.96 [5.21–8.08]	6.59 [4.23–11.17]	NS

Variables are expressed as median and interquartile range. *p* values refer to Wilcoxon test. *NS* non-significant

*Salivary cortisone*: it increased significantly after placebo (5.09 [4.23–6.79] vs 6.48 [5.29–9.00] µg/L, *p* = 0.021), while no variation was observed after diclofenac (Table 2 and Fig. 2).

**Fig. 3:**
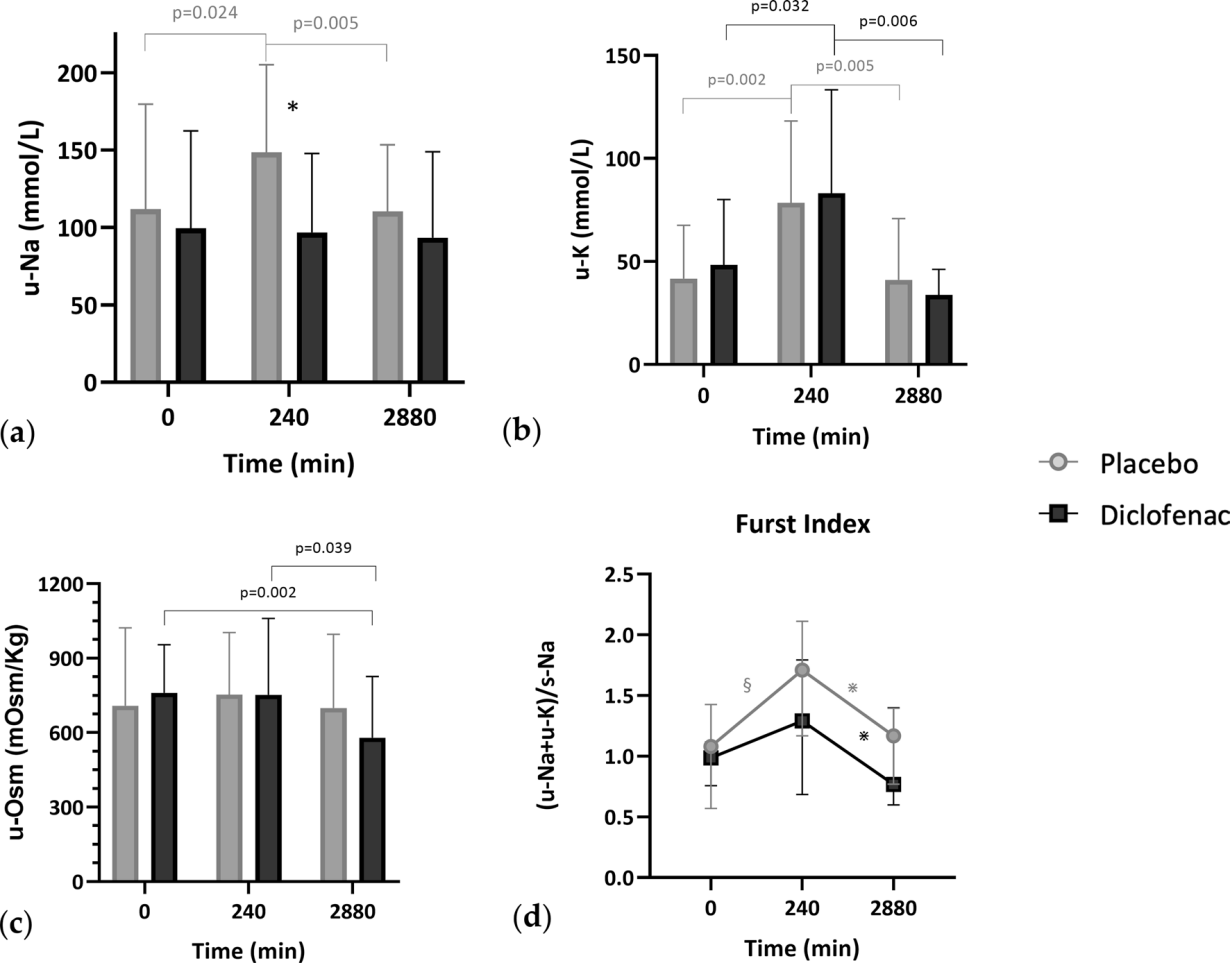
Urine sodium variations (u-Na; **a**), urine potassium variations (u-K; **b**) and urine osmolality (u-Osm; **c**) at baseline (0), at the end of the test (240) and 48 h after (2880) placebo and diclofenac infusion. *p = 0.037 direct comparison between u-Na during placebo and diclofenac at T240 with Student’s* t* test (image **a**); §*p* < 0.001 (T0 vs T240); ⋇*p* = 0.001 (T240 vs T48h); ⋇*p* = 0.042 (T240 vs T48h) (image **d**)

*u-Na*: it increased at the end of the test (T0 vs T240, *p* = 0.024), returning to baseline values after 48 h hours during placebo (T240 vs T48h, *p* = 0.005), whereas it was stable during diclofenac (Table 3 and Fig. 3a).

*u-K*: it showed the same trend in both experimental conditions (*p* < 0.032), increasing at the end of the test and returning to baseline values after 48 h (Table 3 and Fig. 3b).

*u-Osm*: it remained stable during saline infusion, but was significantly (177 ± 5.7 mOsm/Kg) lower 48 h after diclofenac (T0 vs T48h, *p* = 0.002; T240 vs T48h, *p* = 0.039) (Table 3 and Fig. 3c).

**Table 3:** Urinary parameters at different observation times in the two experimental conditions

Variable	T0	T240	*p* value (0–240)	T48h	*p* value (240-48 h)	T0	T240	*p* value (0–240)	T48h	*p* value (240-48 h)
	Placebo Profile					Diclofenac Profile				
u-Na (mmol/L)	111.75 ± 67.95	148.67 ± 56.58	0.024	110.25 ± 43.13	0.005	99.50 ± 63.06	96.92 ± 50.87	NS	93.50 ± 55.49	NS
u-K (mmol/L)	41.73 ± 25.82	78.38 ± 39.82	0.002	41.08 ± 29.75	0.005	48.33 ± 31.74	83.12 ± 50.23	0.032	33.83 ± 12.39	0.006
u-Osm (mOsm/ Kg)	708.17 ± 313.91	753.08 ± 249.98	NS	699.67 ± 296.53	NS	760 ± 194.46	752.00 ± 308.10	NS	579.08 ± 247.41	0.039
Furst Index	1.08 [0.67–1.42]	1.71 [1.19–2.06]	0.002	1.17 [0.86–1.37]	0.002	0.99 [0.78–1.12]	1.29 [0.72–1.78]	NS	0.77 [0.62–1.38]	0.042

Normal variables are expressed as mean ± standard deviation; non-normal variables that can be normalized after logarithmic transformation, are expressed as geometric mean and interquartile range; non-normal and unnormalisable variables are expressed as median and interquartile range. *u-Na* urine sodium; *u-K* urine potassium, *u-Osm* urine osmolality, *NS* non-significant

*Furst index*: being calculated as the urinary electrolytes (u-Na and u-K) to s-Na ratio, it increased at the end of the test during placebo (T0 vs T240, *p* < 0.002) and then went back to baseline values (T240 vs T48h, *p* = 0.002); during diclofenac, it showed a significant reduction only at T48h (*p* = 0.042) (Table 3 and Fig. 3d).

A t-test for paired samples was conducted at every observation time to directly compare each of the urinary variables, only u-Na value at 240’ differed significantly, being higher after placebo than diclofenac (148.67 ± 56.58 vs 96.92 ± 50.87 mmol/L, *p* = 0.037).

*Fluid balance*: regardless of the experimental condition, no changes were observed in reported fluid intake nor in urine output before and after the test.

### BIVA analysis

**Fig. 4:**
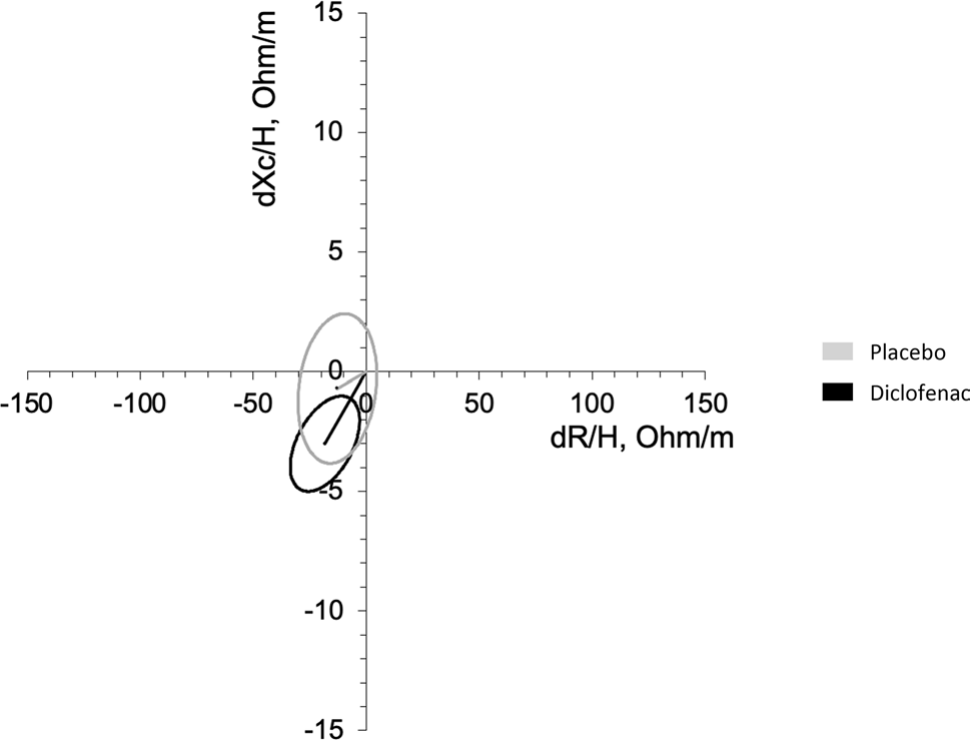
Mean Biavectors’ displacement from T240 to T48h. No differences occurred after placebo (p not significant), but a significant rehydration occurred after diclofenac (*p* < 0.00001) infusion, compared with the paired one-sample Hotelling’s T2 test

*BIVA parameters*: no significant differences were observed when comparing BIVA at T0 and T240 in the two conditions, even though a slight dehydration could be appreciated. A remarkable difference was instead observed when comparing T240 and 48 h: the placebo Biavector remained stable, while the diclofenac one was significantly shifted in the direction of fluid retention (*p* < 0.00001) (Fig. 4).

*TBW*: during placebo, a slight decrease, albeit not significant, was observed while it increased significantly at 48 h (T240 vs T48h, *p* = 0.030). During diclofenac, it decreased at the end of the test (T0 vs T240, *p* = 0.012) and it greatly increased at 48 h (T240 vs T48h, *p* = 0.003), being slightly, but non-significantly, higher than basal values (T0 vs T48h, *p* = 0.056).

*ECW*: it was not affected during placebo, but it increased 48 h after diclofenac (T0 vs T48h, 15.42 ± 1.57 vs 16.47 ± 1.65 L, *p* < 0.001).

*ICW*: it showed a mild non-significant deflection at T240 in both conditions and decreased back to basal values at T48h.

When compared directly, only ECW at T48h was significantly different after placebo or diclofenac (placebo vs diclofenac: 15.93 ± 1.59 vs 16.47 ± 1.65 L, *p* = 0.019) (Supplementary table 1).

## Discussion

Our study demonstrates that diclofenac, acutely and in healthy subjects, induces a significant ECF increase 48 h after infusion, apparently as a main consequence of loco-regional pharmacological effects in the kidney; indeed, our data exclude any significant variation in AVP secretion due to diclofenac administration as evidenced by plasma copeptin determination.

Diclofenac is one of the few NSAIDs that are able to cross the blood brain barrier; it was demonstrated in a murine model that centrally PGs favor AVP release [12] and it is therefore reasonable that such drug may reduce AVP production. However, this evidence comes mainly from outdated studies and the determination of circulating AVP levels is currently not recommended, mainly due to preanalytical sample variability.

In our study, we used the copeptin assay, in consideration of its greater stability and half-life compared to AVP, showing substantial stability in the release of the glycopeptide secreted in equimolar amount with hypothalamic nonapeptide. In both experimental conditions copeptin showed a decreasing trend, likely due to its spontaneous daily fluctuation, which cannot be properly defined as a circadian rhythm [13, 14]. Indeed, it has been described that copeptin reaches higher levels at night and early in the morning from 04:00 am to 06:00 am, which progressively decrease reaching *nadir* between 05:00 pm and 07:00 pm. At the same time, in our study, no peak was registered in copeptin levels, as would be expected if the AVP hypothalamic release was enhanced by diclofenac infusion; moreover, no cases of hyponatremia were reported neither in acute setting nor after 48 h.

As known, SIAD is a potential adverse effect of NSAIDs, but it is also multifactorial, favored by the presence of comorbidities [15]. As a matter of fact, NSAIDs are often used in clinical practice for their analgesic role, and intense pain represents an additional non-osmotic stimulus for AVP secretion. In this context, the effects on the secretory AVP pathway can be enhanced by the concomitant intake of this class of drugs [16]. Finally, it is possible that especially a prolonged use of NSAIDs can induce SIAD, as was previously described after an extended treatment with ketorolac [17], rather than a single acute administration.

Regarding the HPA axis, an in vitro study conducted in 1984 demonstrated that indomethacin, flurbiprofen and diclofenac can increase ACTH release after AVP or CRH stimulus and that this effect does not happen when PGE2 is added [5]. Authors concluded that AVP and CRH increase PGE2 levels in corticotropic cells and that, in turn, PGE2 somehow acts with negative feedback on AVP and CRH action on the hypothalamus. In our study, circulating blood cortisol levels did not change after diclofenac infusion; indeed, they decreased during the test, according to the normal circadian rhythm. Intriguingly, salivary cortisol and cortisone significantly increased only in the night after placebo. Test day likely represented a stressing stimulus for subjects, leading to HPA axis activation so it can be hypothesized that diclofenac mitigates the HPA response to stress.

Moreover, our study provides the first evidence about the lack of effects of diclofenac on other neuroendocrine modulators of the hydro-electrolytic metabolism, such as ANP and ADM. These peptides, however, are not currently measured in routine clinical practice, as more reliable and analytically stable options are available: MR-proANP [18] and MR-proADM [19]. Whereas interactions between diclofenac and MR-proADM had not been explored before, a few data about diclofenac effects on natriuretic peptides were available. Our study partially confirms the finding of Castellani et al. [6] since the drug does not seem to alter MR-proANP circulating levels. Anyway, we did find a possible significant effect on natriuresis.

Indeed, after placebo infusion, u-Na significantly increased compared to diclofenac. The primary PG involved in natriuresis after an increase in renal perfusion pressure is PGE2: it decreases Na reabsorption by inhibiting the Na/K/Cl type 2 transporter in the ascending limb of the loop of Henle [20]. Selective COX-2 inhibitors can occasionally cause Na retention in healthy subjects [21, 22], reducing urinary Na excretion in the first 72 h after administration [21, 23]. More recently, a study conducted in elderly patients in a sodium-controlled diet, demonstrated that both selective and non-selective COX inhibitors reduce Na urinary excretion [24]. Acute Na retention is likely due to COX-2 inhibition, while COX-1 inhibition is responsible for the glomerular filtration rate reduction [23].

In agreement to this, after diclofenac, a non-selective inhibitor with an affinity for COX-2 comparable to that of celecoxib, Na urinary excretion did not increase, as it did after placebo.

Of note, u-K increase at the end of the test was likely due to the slight dehydration that subjects experienced, since K urinary excretion commonly increases in such condition, regardless of its cause [25].

Taken together, u-Na and u-K trends justify the early increase in the Furst index observed only in the placebo setting. This index, helpful in estimating electrolyte-free water clearance, is calculated as the ratio between urinary electrolytes and s-Na; thus, if it results < 1, the subject is excreting free water [26]. Interestingly, our data suggest that excretion of electrolyte-free water is not contrasted in an acute setting by diclofenac, as would be expected if an increase in the activity of the antidiuretic system had happened.

Finally, the significant reduction of u-Osm 48 h after diclofenac is likely due to the restored capacity to eliminate electrolyte-free water in association with a more pronounced rehydration occurred in this setting; as known, AVP physiologically stimulates PGE2 production in the kidney, which acts as a buffer to electrolyte-free water reabsorption induced by the neuropeptide. Moreover, PGE2 increases medullary blood flow and reduces Na/K/Cl type 2 transporter expression on the thick ascending limb of the loop of Henle, thus reducing the medullary osmotic gradient. Therefore, the final effect of PGE2 blockage is an enhancement in electrolyte-free water reabsorption [27].

The Biavector^®^ and Biagram^®^ graph clearly show that fluid distribution was particularly stable during placebo, whereas during diclofenac, after a mild dehydration at the end of the test, a marked rehydration occurred, associated with a significant increase in ECW of approximately 1 L. Considering the previously discussed potential acute Na retention induced by diclofenac and persistent PGE2 inhibition, it can be hypothesized that these mechanisms are partially responsible for the hydro-retentive short-term effect which was observed only after diclofenac administration.

Our study presents some strengths and limitations. To begin with, this study was the first conducted in healthy subjects that thoroughly evaluated neuroendocrine effects of diclofenac on the different systems regulating the hydro-electrolytic balance. The cross-over design and the control with placebo allowed to reduce potential confounding factors and better highlight the effects of the drug. The carry-over effect can be ruled out with a certain confidence given the short half-life of diclofenac and the three weeks gap between the test sessions. Moreover, the most recent and newest technologies were used for the determination of hormones and relative biomarkers. BIVA was integrated in the protocol as it is becoming more and more useful in evaluating hydro-electrolytic balance both in physiologic and pathologic conditions. Even though the renin–angiotensin–aldosterone system (RAAS) was not directly investigated, few outdated studies that investigated the matter suggest that NSAIDs have an inhibitory effect on this system [28, 29], thus the hydro-retentive phenomenon of diclofenac in our experimental setting is unlikely to be caused by a possible RAAS activation. Certainly, the sample size was small; as noted above, the proof-of-concept nature of the study did not allow to calculate the power of the study from which to derive the number of subjects to enroll. Another limit is the evaluation of fluid balance, which is difficult to estimate, especially in a population of working subjects that consumes meal out of home; eventual fluid losses due to physical activity were not taken into consideration. Finally, MR-proANP, MR-proADM, cortisol and ACTH were analyzed less frequently, as it was a secondary analysis on stocked samples and, based on the substantial stability of copeptin levels, the time points were chosen arbitrarily.

## Conclusions

In an acute setting and on healthy subjects, diclofenac determined an increase in body water, in particular ECW, which is evident 48 h after the drug administration. As previously hypothesized in literature, this phenomenon does not seem to be determined by an increase in AVP secretion, but rather by an increase in collecting duct principal cells’ sensibility to the neurohormone and/or by AVP-independent mechanisms. Based on the data from salivary cortisone profiles, a partial inhibitory effect on cortisol secretion can also be hypothesized.

### Supplementary Information

Below is the link to the electronic supplementary material.

Supplementary file 1 Bioimpedance parameters at different observation times in the two experimental conditions. Rz: resistance; Xc: reactance; PhA: phase angle; TBW: total body water; ECW: extracellular water; ICW: intracellular water; NS: non-significant (DOCX 17 KB)

Supplementary file 2 MR-proADM (A) and MR-proANP (C) trend during placebo and diclofenac infusion. The area under the curve (AUC) (B and D) was calculated for each subject and then compared with Student’s t-test. §: p=0.003 for placebo profile (image C). MR-proADM: mid-regional pro-adrenomedullin; MR-proANP: mid-regional pro-atrial natriuretic peptide; ns: non-significant (TIF 1241 KB)

Supplementary file 3 ACTH (A) and cortisol (C) trend during placebo and diclofenac infusion. The area under the curve (AUC) (B) was calculated for each subject and then compared with Student’s t-test. The AUC was not calculated for cortisol as T0 was different in the two conditions (*: p=0.037 direct comparison between placebo and diclofenac with Student’s t-test). ACTH: adrenocorticotropin hormone; ns: non-significant. §: p=0.001 for placebo profile; *: p=0.012 for diclofenac profile (image A); §: p<0.0001 for placebo profile; *: p<0.001 for diclofenac profile (image C) (TIF 1062 KB)

## Data Availability

The datasets generated during and/or analysed during the current study are available from the corresponding author on reasonable request.
