# Supplementary material for: Neuroendocrine response to diclofenac in healthy subjects: a pilot study
Source: J Endocrinol Invest. 2023 May 27;46(12):2617–27. doi: 10.1007/s40618-023-02118-z (PMC10632215; doi:10.1007/s40618-023-02118-z)
Supplement: Supplementary file 1 — Bioimpedance parameters at different observation times in the two experimental conditions. Rz: resistance; Xc: reactance; PhA: phase angle; TBW: total body water; ECW: extracellular water; ICW: intracellular water; NS: non-significant (DOCX 17 KB) [file 40618_2023_2118_MOESM1_ESM.docx]

**Supplementary table 1** Bioimpedance parameters at different observation times in the two experimental conditions.

| **Variable** | **T0** | **T240** | **p value (0-240)** | **T48h** | **p value (240-48h)** | **T0** | **T240** | **p value (0-240)** | **T48h** | **p value (240-48h)** |
| --- | --- | --- | --- | --- | --- | --- | --- | --- | --- | --- |
|  |  | **Placebo Profile** | | | |  | **Diclofenac Profile** | | | |
| **Rz (Ohm)** | 533.35  ±58.97 | 542.57  ±50.88 | NS | 520.43  ±67.00 | NS | 533.33  ±73.21 | 544.88  ±71.00 | 0.017 | 513.48  ±68.79 | 0.005 |
| **Xc (Ohm)** | 62.52  ±5.24 | 62.68  ±9.41 | NS | 61.38  ±4.60 | NS | 62.28  ±6.16 | 64.08  ±7.79 | NS | 59.04  ±6.97 | 0.001 |
| **PhA (°)** | 6.73  ±0.63 | 6.60  ±0.89 | NS | 6.79  ±0.67 | NS | 6.71  ±0.63 | 6.73  ±0.68 | NS | 6.61  ±0.66 | NS |
| **TBW (L)** | 37.07  ±4.68 | 36.58  ±4.10 | NS | 37.73  ±5.22 | 0.030 | 37.41  ±5.07 | 36.85  ±4.81 | 0.012 | 38.32  ±5.23 | 0.003 |
| **TBW%** | 60.93  ±2.81 | 60.33  ±2.75 | NS | 61.88  ±3.67 | NS | 61.37  ±3.73 | 60.70  ±3.94 | 0.047 | 62.28  ±4.55 | 0.023 |
| **ECW (L)** | 15.76  ±1.45 | 15.80  ±1.93 | NS | 15.93  ±1.59 | NS | 15.92  ±1.57 | 15.67  ±1.84 | NS | 16.47  ±1.65 | <0.001 |
| **ECW%** | 42.69  ±2.60 | 43.36  ±3.87 | NS | 42.45  ±2.84 | NS | 42.74  ±2.48 | 42.66  ±2.71 | NS | 43.23  ±2.76 | NS |
| **ICW (L)** | 21.31  ±3.44 | 20.76  ±3.11 | NS | 21.80  ±3.86 | NS | 21.49  ±3.69 | 21.18  ±3.35 | NS | 21.85  ±3.82 | NS |
| **ICW%** | 57.31  ±2.60 | 56.64  ±3.87 | NS | 57.55  ±2.84 | NS | 57.29  ±2.48 | 57.34  ±2.71 | NS | 56.78  ±2.76 | NS |

Rz: resistance; Xc: reactance; PhA: phase angle; TBW: total body water; ECW: extracellular water; ICW: intracellular water; NS: non-significant
