# Supplementary figures and images for: Neuroendocrine response to diclofenac in healthy subjects: a pilot study
Source: J Endocrinol Invest. 2023 May 27;46(12):2617–27. doi: 10.1007/s40618-023-02118-z (PMC10632215; doi:10.1007/s40618-023-02118-z)

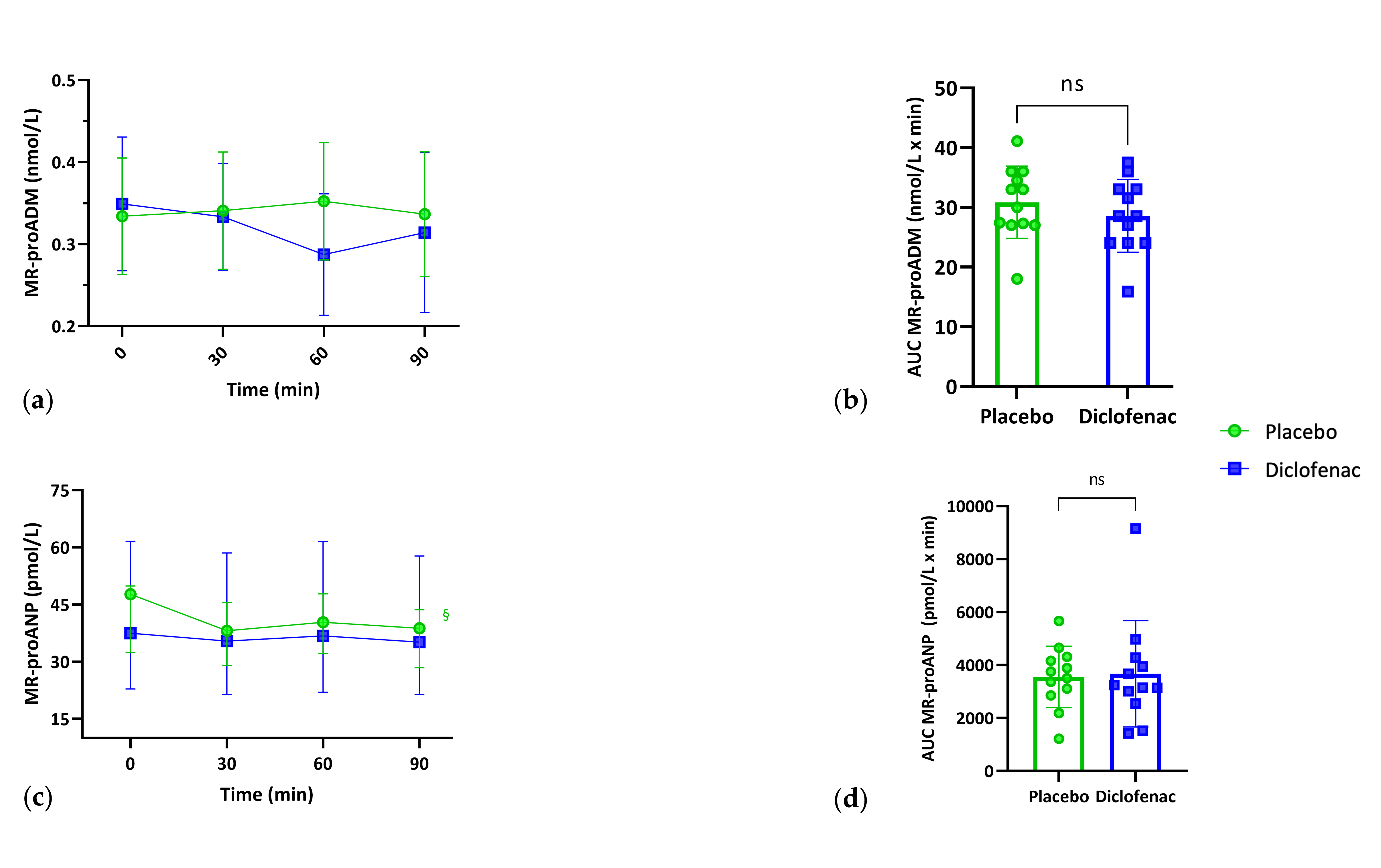

Supplement: Supplementary file 2 — MR-proADM (A) and MR-proANP (C) trend during placebo and diclofenac infusion. The area under the curve (AUC) (B and D) was calculated for each subject and then compared with Student’s t-test. §: p=0.003 for placebo profile (image C). MR-proADM: mid-regional pro-adrenomedullin; MR-proANP: mid-regional pro-atrial natriuretic peptide; ns: non-significant (TIF 1241 KB) [file 40618_2023_2118_MOESM2_ESM.tif]

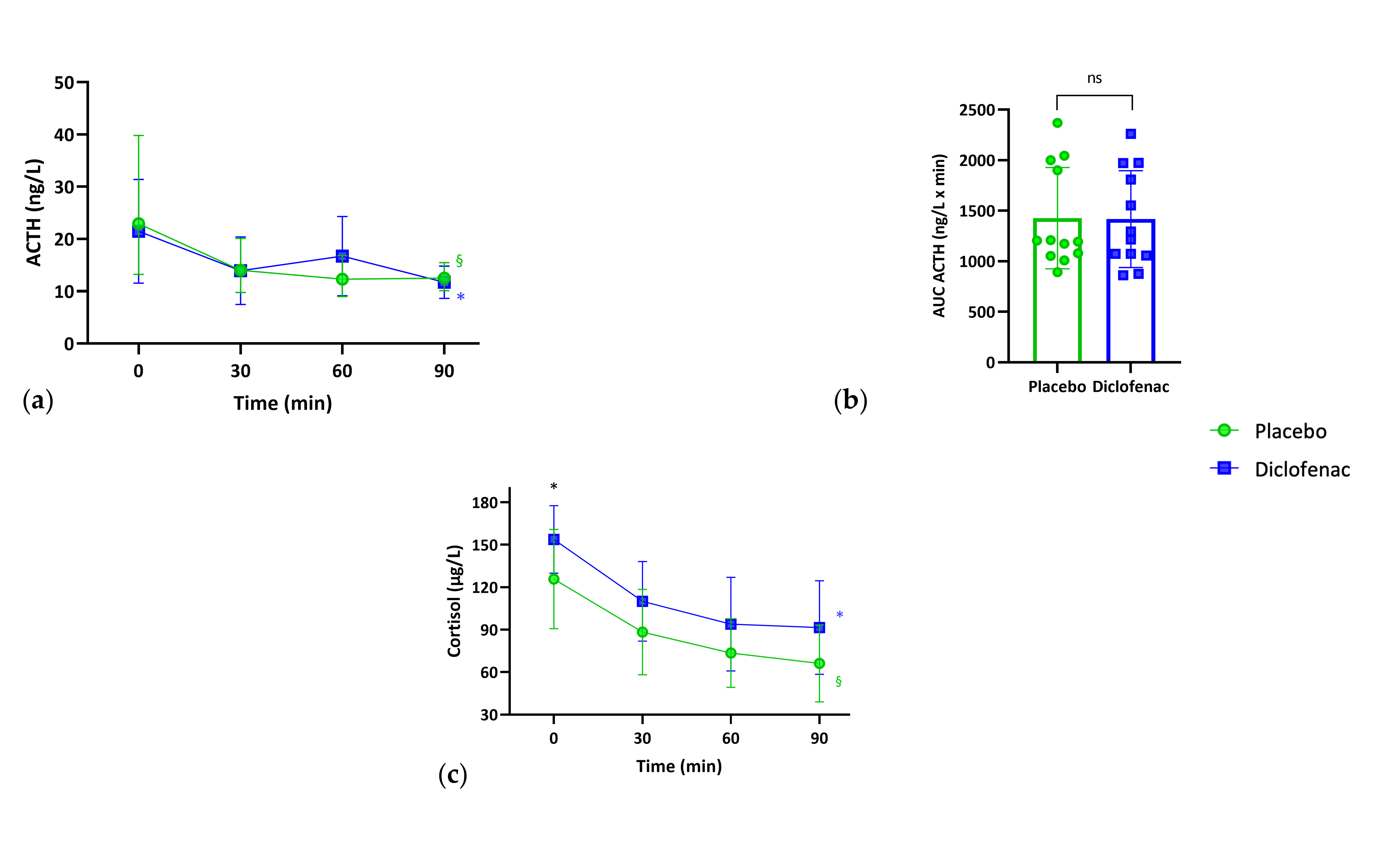

Supplement: Supplementary file 3 — ACTH (A) and cortisol (C) trend during placebo and diclofenac infusion. The area under the curve (AUC) (B) was calculated for each subject and then compared with Student’s t-test. The AUC was not calculated for cortisol as T0 was different in the two conditions (*: p=0.037 direct comparison between placebo and diclofenac with Student’s t-test). ACTH: adrenocorticotropin hormone; ns: non-significant. §: p=0.001 for placebo profile; *: p=0.012 for diclofenac profile (image A); §: p<0.0001 for placebo profile; *: p<0.001 for diclofenac profile (image C) (TIF 1062 KB) [file 40618_2023_2118_MOESM3_ESM.tif]
